# Impact of preoperative patient education on prevention of postoperative complications after major visceral surgery: study protocol for a randomized controlled trial (PEDUCAT trial)

**DOI:** 10.1186/1745-6215-14-271

**Published:** 2013-08-26

**Authors:** Christine Fink, Markus K Diener, Thomas Bruckner, Gisela Müller, Lisa Paulsen, Monika Keller, Markus W Büchler, Phillip Knebel

**Affiliations:** 1 The Study Center of the German Surgical Society, University of Heidelberg, Heidelberg, Germany; 2 Department of General, Visceral and Transplantation Surgery, University of Heidelberg, Im Neuenheimer Feld 110, 69120 Heidelberg, Germany; 3 Psychosocial Care Unit, Department of Surgery, University Hospital, Heidelberg, Germany; 4 Institute of Medical Biometry and Informatics, University of Heidelberg, Heidelberg, Germany

**Keywords:** Patient education, Preoperative education, Postoperative complication, Prevention, Visceral surgery, Cluster randomization

## Abstract

**Background:**

In line with the growing number of surgical procedures being performed worldwide, postoperative complications are also increasing proportionately. Prevention of these postoperative complications is a high medical priority. Preoperative education of patients, including provision of preparatory information about the correct behavior after surgery, could improve the postoperative outcome, but the evidence for this is inconclusive. The aim of the PEDUCAT trial is to evaluate the feasibility and the impact of preoperative patient education on postoperative morbidity, mortality and quality of life in patients scheduled for elective major visceral surgery.

**Methods/design:**

PEDUCAT is designed as a cluster-randomized controlled pilot study. The experimental group will visit a standardized preoperative seminar to learn how best to behave after surgery in addition to being given a standard information brochure, whereas the control group will only receive the information brochure. Outcome measures such as postoperative morbidity, postoperative pain, postoperative anxiety and depression, patient satisfaction, quality of life, length of hospital stay and postoperative mortality will be evaluated. Statistical analysis will be based on the intention-to-treat population. Analysis of covariance will be applied for the intervention group comparison, adjusting for age, center and quality of life before surgery. This is a pilot study to show the feasibility of the concept. Nevertheless, the planned sample size of n = 204 is large enough to show an effect with power of 90% and a significance level of 5%.

**Trial registration:**

German Clinical Trial Register number: DRKS00004226.

## Background

### Rationale

Despite the many advances in medicine, the need for surgical procedures remains high. Each year, an estimated 234 million major surgical procedures are conducted worldwide. Evidence suggests that postsurgical complications occur in at least seven million cases annually, resulting in up to one million deaths [1]. These figures illustrate the tremendous socio-economic burden associated with postoperative morbidity and mortality. The prevention of these postoperative complications is of the highest medical interest and importance. At present, most patients receive information about the best possible postoperative behavior during ward rounds or at the preoperative patient briefing. Due to cost cutting in the healthcare sector, the stay in hospital and the time available for patient consultations are becoming even shorter. Therefore, the impact of standardized preoperative patient education on postoperative outcome could be considerable, but the evidence for this is currently inconclusive. The aim of the PEDUCAT trial is to evaluate the feasibility and to investigate the impact of preoperative patient education on postoperative morbidity, mortality and quality of life in patients scheduled for elective major visceral surgical procedures.

### Preliminary data

Early research, published by Janis in 1958, demonstrated that giving preoperative education to patients is beneficial. He realized that successful emotional inoculation could be achieved in patients facing severe stress by giving them preparatory information containing accurate warnings about what to expect [2]. After Janis had opened up this field of research, numerous studies were conducted to investigate the impact of preoperative education. Hayward showed that patients who had received preoperative information required less analgesia and recovered faster than those who had not [3]. A meta-analysis of 191 studies focusing on how psycho-educational interventions influence recovery showed a positive effect of preoperative patient education on postsurgical pain, psychological wellbeing, anxiety and satisfaction [4]. Ronco *et al*. published a systematic review from 2004 to 2010 evaluating a total of 19 studies involving 3,944 patients. Frequently analyzed outcomes were anxiety, knowledge, pain and length of hospital stay. The objective knowledge was the only positive outcome influenced by patient education [5]. A study by Costa *et al*. showed that inadequate preoperative preparation of patients and lack of information with regard to their postoperative process was associated with unexpected pain and fatigue [6]. Breemhaar *et al*. argued that the provision of education and support had a positive effect on surgical patients’ physical and psychological wellbeing before and after surgery because education and support maintain or increase the patient’s feelings of control [7].

In summary, providing preoperative education might improve patients’ understanding and greatly alleviate their anxieties and fears with regard to the surgical experience, helping them to remain calm and face the situation in a positive way. In a review published in 1998, however, Shuldham showed that most of the studies conducted from the 1950s to the 1980s did not meet today’s methodological standards for clinical studies owing to lack of blinding, unclear randomization protocols, lack of sample homogeneity and insufficient sample size [8]. Frequently, heterogeneous groups of patients undergoing various types of operations were used. However, indices such as pain may vary widely depending upon the surgical procedure. In a second review, Shuldham found no demonstrable benefit from preoperative teaching of the patient having coronary artery bypass surgery and further research was suggested [9]. In a randomized controlled trial led by the same author, patients with bypass surgery were again investigated. No significant impact of patient education on anxiety, pain or depression was found. However, the length of hospital stay differed significantly in the patients who were given additional presurgical information. Shuldham *et al*. concluded that their results demonstrated that no significant benefit was to be gained from preoperative education [10].

In conclusion, the impact of preoperative patient education on the postoperative outcome remains unclear. Hitherto, studies investigating preoperative education have focused mainly on endpoints such as stress, anxiety, satisfaction and quality of life. The PEDUCAT trial will evaluate outcome measures such as postoperative morbidity (for example, pneumonia, deep vein thrombosis, pulmonary embolism, burst abdomen, in-hospital falls) and mortality in visceral surgery. Additionally, most studies conducted so far are analyzing the impact of patient education on postoperative complications after minor surgical interventions, such as laparoscopic cholecystectomy, inguinal hernia repair or gastric band surgery [11-13]. As distinguished from the studies performed until now the PEDUCAT trial is focusing on prevention of postoperative complications after major visceral surgery, for instance multivisceral resection, pancreaticoduodenectomy, esophagectomy, or rectal resection. After these major interventions a higher incidence of postoperative complications is expected and subsequently the impact of a preoperative patient education could be more evident.

### Objective

The objective of the PEDUCAT trial is to evaluate the feasibility of preoperative education and its impact on postoperative morbidity, mortality, postoperative pain, perioperative anxiety and depression, quality of life and length of hospital stay in 204 patients.

## Methods/design

### Study population and eligibility criteria

Patients scheduled for an elective major visceral surgical procedure at the Department of General, Visceral and Transplantation Surgery, University of Heidelberg, Germany will be recruited. A major procedure is defined as a surgical intervention with a planned procedure time equal to or greater than 180 minutes. The complex surgical interventions involve for instance multivisceral resection, pancreaticoduodenectomy, total pancreatectomy, partial or full gastrectomy, transhiatal or transthoracic esophagectomy, major hepatectomy, lower anterior rectal resection or abdominoperineal resection. Moreover, all participants aged 18 years or older who have given written informed consent will be eligible. The included patients have to be capable of fully understanding the contents that are explained during the seminar and illustrated in the information brochure. Furthermore, the participants have to be able to read and understand the informed consent form. Patients with impaired mental state, patients deemed to have insufficient understanding of the German language, patients who cannot participate due to physical constraints or infections requiring isolation, and patients who have already taken part in the preoperative education program in Heidelberg will be excluded from the PEDUCAT trial.

### Sample size

**Figure 1:**
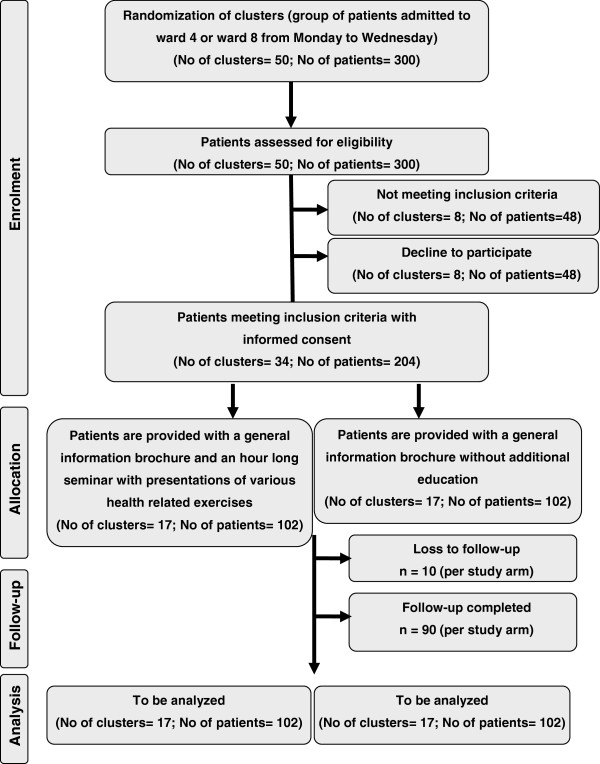
Flow chart of the PEDUCAT trial.

A total of 300 patients (50 clusters) will be randomized and screened for eligibility. The number of patients meeting the inclusion criteria will be 204 (34 clusters). One hundred and two patients (17 clusters) per group will be analyzed (see Figure 1).

### Type of trial

The PEDUCAT trial is a single-center, explorative, cluster-randomized, controlled surgical pilot study with two parallel study groups.

### Recruitment and trial timeline

The estimated time frame for randomization of 204 eligible patients is 34 weeks. The total duration of the trial is expected to be 13 months, including analysis. Recruitment of participants started in October 2012 as a single-center trial. Patients are followed up for 30 days after surgery. The actual overall duration or recruitment time may differ.

### Randomization

Two wards of the Department of General, Visceral and Transplantation Surgery, University of Heidelberg, each with 36 beds, are participating in the trial. The patients are all comparable with regard to their diagnosis, planned surgery and baseline data. Patients admitted from Monday to Wednesday on each of the two wards are defined as a cluster. There will be two clusters per week. In order to prevent selection bias and to achieve comparable groups for known and unknown risk factors, randomization will be performed on a block basis with 1:1 allocation. Subjects are assigned to either group A or group B based on a computer-generated randomization list prepared by the Institute of Medical Biometry and Informatics, University of Heidelberg, Germany. The randomization list is in the charge of an independent member of staff of the Clinical Study Center Surgery, Department of General, Visceral and Transplantation Surgery, University of Heidelberg who discloses the randomization of the two wards every second week. The investigators are informed, and the wards are updated about the randomization result.

## Interventions

### Experimental intervention

Patients take part in a one-hour-long seminar with presentations of various health-related exercises. This patient education takes place one day before surgery and is carried out by qualified nursing staff. Currently, 17 nurses are involved in the program and, on average, around 800 patients take part each year. During the seminar the patients receive information about the options for acute pain therapy (for example, patient-controlled analgesia (PCA), peridural anesthesia (PDA) or oral painkillers) as well as different coping strategies (for example, autogenic training, progressive muscle relaxation). The patients learn that pain therapy is recommended if the visual analogue pain scale (VAS) score exceeds a certain value. Moreover, it is pointed out that adequate pain therapy is essential for prevention of pulmonary complications such as pneumonia and for the treatment of atelectasis. Patients are introduced to practical exercises with Flutter® or Triflo®. The recommended frequency and intensity of various breathing exercises are also mentioned. Furthermore, the importance of postoperative mobilization with regard to preventing thrombosis is pointed out. Physical exercises and the correct use of compression stockings to reduce the risk of thrombosis are also demonstrated. Special methods such as careful coughing are taught to prevent burst abdomen. In addition, ways to get out of bed with the least amount of pain and without increased abdominal pressure are demonstrated. Patients will be encouraged to practice the out-of-bed mobilization preoperatively. Several risk factors for postoperative in-hospital falls are discussed, for instance the combination of certain drugs taken during the hospital stay. The patients are reminded that for a certain time after surgery they should only get up while attended by a nurse. Exercises to stimulate the circulation are introduced which also serve to prevent thrombosis. Moreover, the patients are informed about the importance of avoiding muscle degeneration and muscle stimulation. Verbal instructions are backed up by a PowerPoint presentation. A manual for the instructors was created to ensure standardization of the seminar. Additionally, patients are provided with a 48-page information brochure in which every topic covered in the seminar is briefly explained and illustrated.

### Control intervention

Patients are only provided with the general information brochure mentioned above, with no additional education or exercises.

### Risks

No additional risks for study patients are anticipated.

### Outcomes

● Postoperative morbidity: frequency and type of postoperative complication after surgery up to postoperative day 30 is recorded. Complications are graded according to the Clavien-Dindo Classification (Table 1). For definition of the endpoints of postoperative morbidity (pneumonia, deep vein thrombosis, pulmonary embolism, burst abdomen, in-hospital falls) see Table 2.

● Postoperative pain: a modified version of the validated Brief Pain Inventory (BPI) is used in this study. It contains three severity items (worst pain, pain on average, pain right now) and five interference items (walking ability, mood, sleep, relation to others, ability to concentrate) [15].

● Perioperative anxiety and depression: the Hospital Anxiety and Depression Scale (HADS) is used to determine the level of anxiety and depression that a patient is experiencing [16].

● Patient satisfaction: is evaluated with an unvalidated questionnaire comprising seven items.

● Quality of life: the patient’s quality of life is assessed by means of the SF-12 questionnaire.

● Length of hospital stay: the time from day of operation until discharge from hospital.

● Postoperative mortality: death from any cause within 30 days after major visceral surgery.

**Table 1:** **The Clavien-Dindo Classification of surgical postoperative complications **[14]

**Grade**	**Definition**
I	Any deviation from the normal postoperative course without the need for pharmacological treatment or surgical, endoscopic, and radiological interventions. Allowed therapeutic regimens are: drugs as antiemetics, antipyretics, analgesics, diuretics, electrolytes, and physiotherapy. This grade also includes wound infections opened at the bedside
II	Requiring pharmacological treatment with drugs other than such allowed for grade I complications. Blood transfusions and total parenteral nutrition are also included
III	Requiring surgical, endoscopic or radiological intervention
IIIa	Intervention not under general anesthesia
IIIb	Intervention under general anesthesia
IV	Life-threatening complications requiring IC/ICU management
IVa	Single organ dysfunction (including dialysis)
IVb	Multiorgan dysfunction
V	Death of a patient

**Table 2:** Definition of the endpoints of postoperative morbidity

1. Pneumonia	At least three of the four following signs:
	• fever, defined as oral or tympanic temperature > 37.5°C or rectal temperature > 38.5°C
	• purulent tracheal sputum production/secretion or change in sputum character
	• total peripheral white blood cell (WBC) count > 12 g/L or WBC < 4.5 g/L or > 15% immature neutrophils (bands), regardless of total peripheral WBC count
	• increased plasma or serum C-reactive protein (CRP) level as shown by a level of at least twice the upper limit of the hospital normal range
	and
	chest X-ray or CT scan findings (anterior-posterior (ap) or posterior-anterior (pa) and, if possible, lateral views) in agreement with the clinical diagnosis of bacterial pneumonia, that is, the appearance of new, progressive pulmonary infiltrate(s) attributable to infectious etiology
2. Deep vein thrombosis	clinical evidence (for example, painful, swollen, warm, livid leg) of a previously unknown thrombosis located in a deep leg or pelvic vein with radiological confirmation by duplex sonography or CT-angiography
3. Pulmonary embolism	clinical suspicion of pulmonary embolism (for example, tachycardia, dyspnea) confirmed by spiral CT or lung perfusion scintigraphy
4. Burst abdomen	postoperative absence of continuity of the abdominal fascia in combination with wound dehiscence requiring reintervention
5. Falls	in-hospital falls

The frequency and type of postoperative complications will be evaluated on days 2, 7 and 30 after surgery. Postoperative pain will be documented at baseline and on days 2 and 7 after surgery with a modified validated version of the BPI. Perioperative anxiety and depression will be investigated with the HADS questionnaire at baseline and on days 7 and 30 after operation. Quality of life will be assessed using the SF-12 questionnaire at baseline and at 30 days postoperatively. Additionally, the length of hospital stay and the postoperative mortality will be evaluated (Table 3).

**Table 3:** Study visits of the PEDUCAT trial

**Documentation**	**Visit 1**	**Visit 2**	**Visit 3**	**Visit 4**	**Visit 5**
	**Screening**	**2 days after surgery**	**7 days after surgery**	**30 days after surgery**	**End of study**
Baseline data, demographics	X				
Eligibility criteria	X				
Randomization	X				
Assessment of complications and safety		X	X	X	X
Morbidity, mortality, length of stay		X	X	X	X
Postoperative pain (BPI)	X	X	X		
Perioperative anxiety and depression (HADS)	X		X	X	
Patient satisfaction			X		
Quality of life (SF-12)	X			X	

### Safety evaluation and reporting of adverse events

Adverse events will not be documented in the PEDUCAT trial unless they fulfill one of the criteria for seriousness (a serious adverse event (SAE) is an event that results in death, is immediately life-threatening, requires or prolongs hospitalization, or results in persistent or significant disability or incapacity). Only SAEs of grade IV or V according to the Clavien-Dindo Classification (see Table 1) need to be reported within the PEDUCAT trial. Moreover, the following complications do not require separate SAE reporting, since they will be documented separately as endpoints in the case report form: pneumonia, deep vein thrombosis, pulmonary embolism, burst abdomen, falls, and mortality. From the day on which the patient gives signed informed consent until the regular end of the trial at 30-day follow-up or until premature withdrawal of the patient, all SAEs must be documented on an SAE form. This form identifies the trial subject and the attending physician and describes the SAE (event, beginning, intensity, duration, severity, outcome, causal relation to the trial intervention, treatment/interventions undertaken); it is dated and signed by the attending physician.

## Statistical methods

### Sample size calculation

This is a pilot study. Therefore, no sample size calculation was performed. A sample size of 102 patients per study group seems sufficient to test the feasibility of patient recruitment and cluster randomization, assuming a cluster size of six patients.

### Statistical analysis

All endpoints will be analyzed descriptively by tabulation of the measures of the empirical distributions. Depending on the scale level of the variables, means, standard deviations, medians, and first and third quartiles, as well as either minimum and maximum or absolute and relative frequency, will be reported. Additionally, Kaplan-Meier analysis will be performed for postoperative mortality. Descriptive *P*-values of the corresponding statistical tests comparing the treatment groups will be given, together with the associated 95% confidence intervals. Because of the cluster randomization, the analyses will involve a multilevel regression approach with patients at level one and education cluster at level two. When appropriate, graphical methods will be used to visualize the findings. The homogeneity of the treatment groups will be described by comparison of the demographic data and the baseline values.

### Withdrawals

Patients give written informed consent to treatment in the trial as well as data collection at the pre-specified study visits. Early trial termination may occur, if patients die or are lost to follow-up. Furthermore, patients are free to withdraw from the trial at any time without providing a specific reason. Patients may completely or partially rescind consent. Patients, who partially rescind consent are those who do not wish to receive any further treatment within the trial but are willing to participate in subsequent study visits and assessments (follow-up). These patients remain in the follow-up assessments and the data obtained are included in the final analyzes. Patients who withdraw consent completely wish neither further treatment within the trial nor further follow-up assessments within the trial.

### Trial organization, data management and monitoring

Coordination and data management of the PEDUCAT trial is performed by the Clinical Study Center Surgery (KSC), Department of General, Visceral and Transplantation Surgery, University of Heidelberg. Statistical analysis is performed by the Institute of Medical Biometry and Informatics (IMBI). The clinical monitoring will be done by the Study Center of the German Society for Surgery (SDGC) according to approved standard operating procedures (SOPs). The PEDUCAT study protocol was written in accordance with the SPIRIT statement [17].

### Ethical and legal aspects

This study is accomplished according to the Helsinki Declaration., the principles of the Good Clinical Practice (ICH-GCP) guidelines (E6) and the Federal Data protection Act. The study protocol was approved by the ethics committee of the University of Heidelberg.

### Subject information and informed consent

Before being admitted to the trial, the patient must consent to participate after the nature, scope, and possible consequences of the clinical trial have been explained in a form understandable to him or her. After reading the informed consent document, the patient must give consent in writing. The investigator will not undertake any measures specifically required only for the clinical trial until valid consent has been obtained.

## Trial status

Recruiting.

## Abbreviations

PCA: Patient-controlled analgesia; PDA: Peridural anesthesia; VAS: Visual pain scale; BPI: Brief pain inventory; HADS: Hospital anxiety and depression scale; SAE: Serious adverse event.

## Competing interests

The authors declared that they have no competing interests.

## Authors’ contributions

CF, MKD, PK, MWB, TB and MK participated in the development (sample size calculation, protocol and funding application) of the trial. CF, PK, GM and LP contributed to the implementation (submission to ethics committee, data management, monitoring and patient enrollment). TB, MKD and PK performed the data handling and statistical analysis. CF, MKD, TB, GM, LP, MK, MWB and PK helped to draft and to review the paper. All authors read and approved the final manuscript.
